# Rat Middle Cerebral Artery Occlusion Is Not a Suitable Model for the Study of Stroke-Induced Spontaneous Infections

**DOI:** 10.1371/journal.pone.0099169

**Published:** 2014-06-12

**Authors:** Mireia Campos-Martorell, M Ángeles Montero, Mar Hernández-Guillamón, Anna Rosell, Javier Gomis, David Salat, Lidia García-Bonilla, Joan Montaner

**Affiliations:** 1 Neurovascular Research Laboratory, Institut de Recerca Vall d'Hebron, Universitat Autònoma de Barcelona, Barcelona, Spain; 2 Department of Anatomic Pathology, Hospital Vall d' Hebron, Barcelona, Spain; 3 Infectious Diseases Research Laboratory, Institut de Recerca Vall d'Hebron, Universitat Autònoma de Barcelona, Barcelona, Spain; 4 Neurovascular Unit, Department of Neurology, Universitat Autònoma de Barcelona, Hospital Vall d' Hebron, Barcelona, Spain; INSERM U894, Centre de Psychiatrie et Neurosciences, Hopital Sainte-Anne and Université Paris 5, France

## Abstract

**Background:**

Infections related to stroke-induced immunodepression are an important complication causing a high rate of death in patients. Several experimental studies in mouse stroke models have described this process but it has never been tested in other species such as rats.

**Methods:**

Our study focused on the appearance of secondary systemic and pulmonary infections in ischemic rats, comparing with sham and naive animals. For that purpose, male Wistar rats were subjected to embolic middle cerebral artery occlusion (eMCAO) or to transient MCAO (tMCAO) inserting a nylon filament. Forty-eight hours after ischemia, blood and lung samples were evaluated.

**Results:**

In eMCAO set, ischemic rats showed a significant decrease in blood-peripheral lymphocytes (naive = 58.8±18.1%, ischemic = 22.9±16.4%) together with an increase in polymorphonuclears (PMNs) (naive = 29.2±14.7%, ischemic = 71.7±19.5%), while no change in monocytes was observed. The increase in PMNs counts was positively correlated with worse neurological outcome 48 hours after eMCAO (r = 0.55, p = 0.043). However, sham animals showed similar changes in peripheral leukocytes as those seen in ischemic rats (lymphocytes: 40.1±19.7%; PMNs: 51.7±19.2%). Analysis of bacteriological lung growth showed clear differences between naive (0±0 CFU/mL; log10) and both sham (3.9±2.5 CFU/mL; log10) and ischemic (4.3±2.9 CFU/mL; log10) groups. Additionally, naive animals presented non-pathological lung histology, while both sham and ischemic showed congestion, edema or hemorrhage. Concordant results were found in the second set of animals submitted to a tMCAO.

**Conclusions:**

Inflammatory and infection changes in Wistar rats subjected to MCAO models may be attributed not only to the brain ischemic injury but to the surgical aggression and/or anaesthetic stress. Consequently, we suggest that stroke-induced immunodepression in ischemic experimental models should be interpreted with caution in further experimental and translational studies, at least in rat stroke models that entail cervicotomy and cranial trepanation.

## Introduction

Stroke not only produces local brain injury but also causes a systemic inflammatory response followed by an immunodepression process which predisposes patients to infections. This immunodepression state is thought to be induced through a sympathetic nervous system over-action which contributes to an increase of catecholamines and glucocortocoids [1]. Thus, the prolongation of this immune suppression may increase the chance of infection [2], predisposing stroke patients to pneumonia and sepsis [3]. On the other hand, it is well known that stroke prognosis depends on secondary complications incidence [4], recognizing infection as an independent risk factor for adverse outcome [3], [5]. However, the precise mechanisms by which this association operates remain poorly understood. The incidence and prognostic impact of infections have been recently evaluated in a meta-analysis on this subject, which concluded that infections complicated acute stroke in 30% of patients [3]. This high incidence on stroke patients seems to be a result of an impaired immune function [6], [7] complicated with stroke-facilitated aspiration, due to patient dysphagia, immobilization and the need of invasive procedures like insertion of intravenous lines and urinary catheters [8]. Pneumonia is the most common infection [3], [9], [10] and it is the leading cause of death within the first 48 hours after stroke [11], [12].

Experimental studies with different animal models have been crucial in understanding several aspects related to the physiopathology of the infection during the acute phase of stroke. Some studies in mouse models of cerebral ischemia (suture MCAO model) have demonstrated that nervous system damage results in spontaneous bacterial infections because of a deterioration of cell-mediated immune responses [8], [13], [14]. Others have shown that when a harmless concentration of *Streptococcus peumoniae* is intranasally applied, only mice with stroke shift to severe infection [15]. However, studies of stroke-induced immunodepression and spontaneous infections associated with brain ischemia have not been previously reported in rats. Therefore, we aimed to study secondary systemic and respiratory tract infections in rats using the MCAO model induced by blood-clot administration in rats, the model that best resembles clinical stroke [16].

## Materials and Methods

### Ethics statement

All procedures were approved by the Animal Ethics Committee of the Vall d'Hebron Research Institute (02/09 and 58/13 CEEA) and were conducted in compliance with the Spanish legislation and in accordance with the Directives of the European Union. In all experiments, male Wistar rats (weighing 275 to 310 g; Charles River Laboratories) were used. Rats were kept in a climate-controlled environment on a 12-hour light/12-hour dark cycle. Food and water were available ad libitum during all experimental process and all efforts were made to minimize suffering.

### Embolic MCAO model

Infarction in the territory of the middle cerebral artery (MCA) was induced by embolic occlusion (embolic MCAO) as previously described [17]. Animals were anesthetized under spontaneous respiration with 2% isoflurane (Abbot Laboratories, Kent, UK) in oxygen during surgery and body temperature was maintained at 37°C. Arterial blood from a donor rat was withdrawn to form 2 clots (length: 1.5 cm; diameter: 0.3 mm) and they both were used for embolization of the right MCA. Cranial trepanation was performed the day before MCAO surgery to attach a laser–Doppler probe (Moor Instruments, Devon, UK) and monitor regional cerebral blood flow. Only animals that exhibited a reduction >75% in regional cerebral blood flow during MCAO were included in the study. Sham animals were submitted to both trepanation and surgery procedures, but no blood clots were injected into arteries. Three doses of analgesia (magnesic metamizol) were administrated just after cranial trepanation, MCAO surgery and 24 hours after it. All animals were euthanized 48 hours after the surgery. Naive animals were not submitted to any procedure and were sacrificed the same day as ischemic and sham ones.

A total of 59 rats were used for this study. Nine of them were naive rats, 13 were sham and 37 were submitted to embolic MCAO. Eleven of the ischemic animals were excluded after applying the following criteria: inappropriate occlusion of the MCA after embolization (n = 6); spontaneous reperfusion within the next ten minutes after occlusion (n = 3) or sudden death during the surgery process (n = 2). Blood samples were drawn through transcardiac puncture 48 hours after embolic MCAO or after sham surgery. Afterwards, rats were transcardially perfused with sterile PBS and lungs were carefully removed in aseptic conditions for posterior evaluation. From the total of 26 rats submitted to embolic MCAO and included in the study, 10 of them died before the experimental protocol finished (eight during the first 24 hours and two of them between 24 and 48 hours after the occlusion). No animals died in the sham group.

### Intraluminal tMCAO model

In order to ratify our findings, experiments were repeated in a new set of rats submitted to a transient MCAO model using an intraluminal filament as described previously [18]. In brief, after the surgical exposure of the bifurcation of the external carotid artery and the internal carotid artery on the right side, a silicone-coated nylon monofilament (Doccol Corporation, reference number: 403723PK10) was introduced to occlude the MCA. After occlusion, animals were allowed to recover from anesthesia. Reperfusion was induced 90 minutes later and to that end, animals were re-anaesthetized. Only animals that exhibited a reduction >75% in regional cerebral blood flow after filament placement and a recovery of >75% after filament removal were included in the study. Sham-operated animals were submitted to the same processes except for the filament introduction. Naive animals were not submitted to any procedure and were sacrificed the same day as ischemic and sham ones. Analgesia protocol and methods employed for sample collection were exactly the same as the ones used in the previous rat set submitted to an eMCAO model.

From the total of 10 rats submitted to tMCAO, two of them were excluded due to bad reperfusion and another one died before the experimental protocol finished. No animals died in the sham group.

### Infarct volume and neurological deficit evaluation

Infarct volume was measured using 2,3,5- triphenyltetrazolium chloride (TTC, Sigma-Aldrich) staining as described [19]. TTC images were captured using a Cano Scan 4200F and infarct volume was measured using Image J software by integration of infarcted areas. Infarct volume data was expressed as a percentage of the ipsilateral hemisphere and edema was evaluated taking into account the following equation: edema = (infarct volume×contralateral volume)/ipsilateral volume.

Rats were assessed using a 9-point neurological deficit scale, as previously described [18]. Four consecutive tests were conducted: (I) spontaneous activity (moving and exploring = 0, moving without exploring = 1, no moving or moving only when pulled by the tail = 2); (II) left drifting during displacement (none = 0, drifting only when elevated by the tail and pushed or pulled = 1, spontaneous drifting = 2, circling without displacement, or spinning = 3), (III) parachute reflex (symmetrical = 0, asymmetrical = 1, contralateral forelimb retracted = 2), and (IV) resistance to left forepaw stretching (stretching not allowed = 0, stretching allowed after some attempts = 1, no resistance = 2). Neurological score was assessed in a blinded manner at 90 minutes, 24 hours and 48 hours after occlusion.

### Flow cytometry

Blood-leukocyte populations (monocytes, polymorphonuclears (PMNs) and lymphocytes) were evaluated by flow cytometry technique. With this aim, 500 µl of blood were incubated with Ammonium-chlorid-kaliumhydrogencarbonat (ACK) buffer (room temperature, 5 minutes) to lysate erythrocytes and later the pellet was rinsed with Hanks' balanced salt solution - Hepes (HBSS-Hepes, 500 g, 5 minutes at 4°C). The same process (lysis-cleanliness-centrifugation) was repeated thrice. Cells were resuspended in 100 µl of FACS buffer, blocked with 0.25 µg purified mouse anti-Rat CD32 (eBioscience, San Diego, Ca, USA) on ice for 10 minutes and stained with 0.4 µg anti-Granulocyte Marker-PE (GrM-PE) (eBioscience) and 1 µg anti-Rat CD11b- FITC (eBioscience) antibodies for 20 minutes on ice. A specific antibody to detect cell viability, To-Pro-3 (eBioscience) was used to assess samples preservation. A total of ten-thousand events per sample were acquired using FacScalibur (Becton Dickinson, USA). To discriminate leukocyte types, the following populations were defined: PMNs were CD11b-FITC^+^ and GrM-PE^+^; monocytes CD11b-FITC^+^ and GrM-PE^-^ and lymphocytes CD11b-FITC^-^ and GrM-PE^-^. Data were analyzed with FCS Express version 3 (De Novo Software, USA) and expressed as percentage of total leukocytes.

### Lung histology

Lungs were carefully removed and separated into right and left lung under sterile conditions. Left lung was immersed in formaldehyde 4% (Sigma, St. Louis, MO, USA) for 48 hours, embedded in paraffin wax and sectioned with a microtome in 4 µm thick sections. Hematoxilyn & Eosin (HE) stain was carried out to perform lung tissue histological evaluation by an investigator blinded for study group.

### Microbiological analysis

Lung imprints were performed pressing 4 times on blood-agar plates' surface with a piece of sterile right lung. Blood was withdrawn by cardiac puncture in sterile conditions and lungs were collected, minced and homogenized also under sterile conditions. One-hundred µL of all specimens were serially diluted in sterile PBS up to 200 µL of final solution, which was plated onto blood agar plates (Biomérieux, Marcy l'Etoile, France). After 24 and 48 hours of incubation at 37°C, agar plates were analyzed for growth colonies. Results of lung imprints were expressed as the number of observed colonies. Results of both blood and lung homogenate were expressed as a logarithmic function (log10).

### CXCL-1 measurement

For the measurement of blood CXCL-1 cytokine, a simple ELISA kit was purchased from R&D systems and used according to the manufacturer's protocol.

### Statistical analysis

Data were analyzed using GraphPad Prism_v5 software. Statistical significance for intergroup differences was tested by Student's t-test and ANOVA followed by Bonferroni post hoc test for parametric data. For non-parametric data, Mann Whitney and Kruskal-Wallis test followed by Dunn's Multiple Comparison test was performed. Correlations between parameters were tested by Pearson (parametric data) or Spearman tests (non- parametric data). For parametric data, bars represent mean± SD and for non- parametric data, box plots represent median (Interquartil range). A *p* value<0.05 was considered statistically significant at a 95% confidence level.

## Results

### Infarct volume and neurological deficit

Infarct volumes of a subgroup of rats submitted to an eMCAO were calculated and the mean was 40.62±10.09% (n = 6), whereas no infarct lesion was observed in sham animals in any case (n = 13). Mean neurological score at 48 hours was 4.43±2.9 for ischemic animals and 0.28±0.82 for sham animals.

### Changes in peripheral leukocytes count are correlated with neurological outcome after stroke

**Figure 1:**
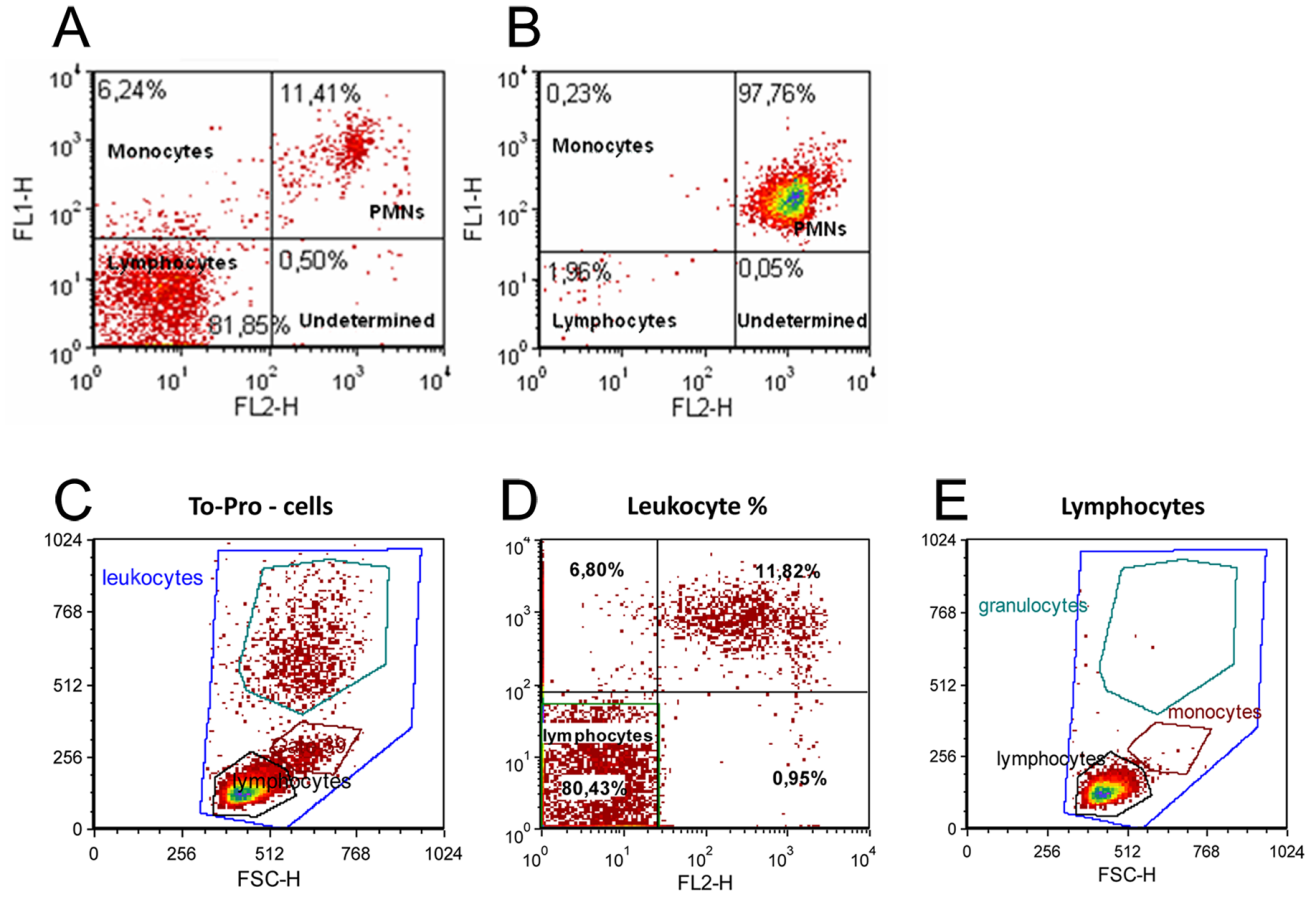
On the upper row: cytometry plots of representative (A) naive and (B) ischemic rats with high neurological score. On the lower row: Strategy used for Lymphocyte identification. (C) Leukocytes distribution considering their granularity (SSC-H) and their size (FSC-H) (D) Leukocyte distribution according to antibody's labelling. FL1-H represents Fit-C and FL2-H, PE. As shown, lymphocytes are CD11b-FITC^-^ and GrM-PE^-^ (E) Lymphocytes view after gating them from graph.

As shown in figures 1A and 1B, striking differences were detected through flow cytometry analysis among animal groups. Leukocyte subpopulations could be properly separated and lymphocytes clearly distinguished (Figure 1C).

**Figure 2:**
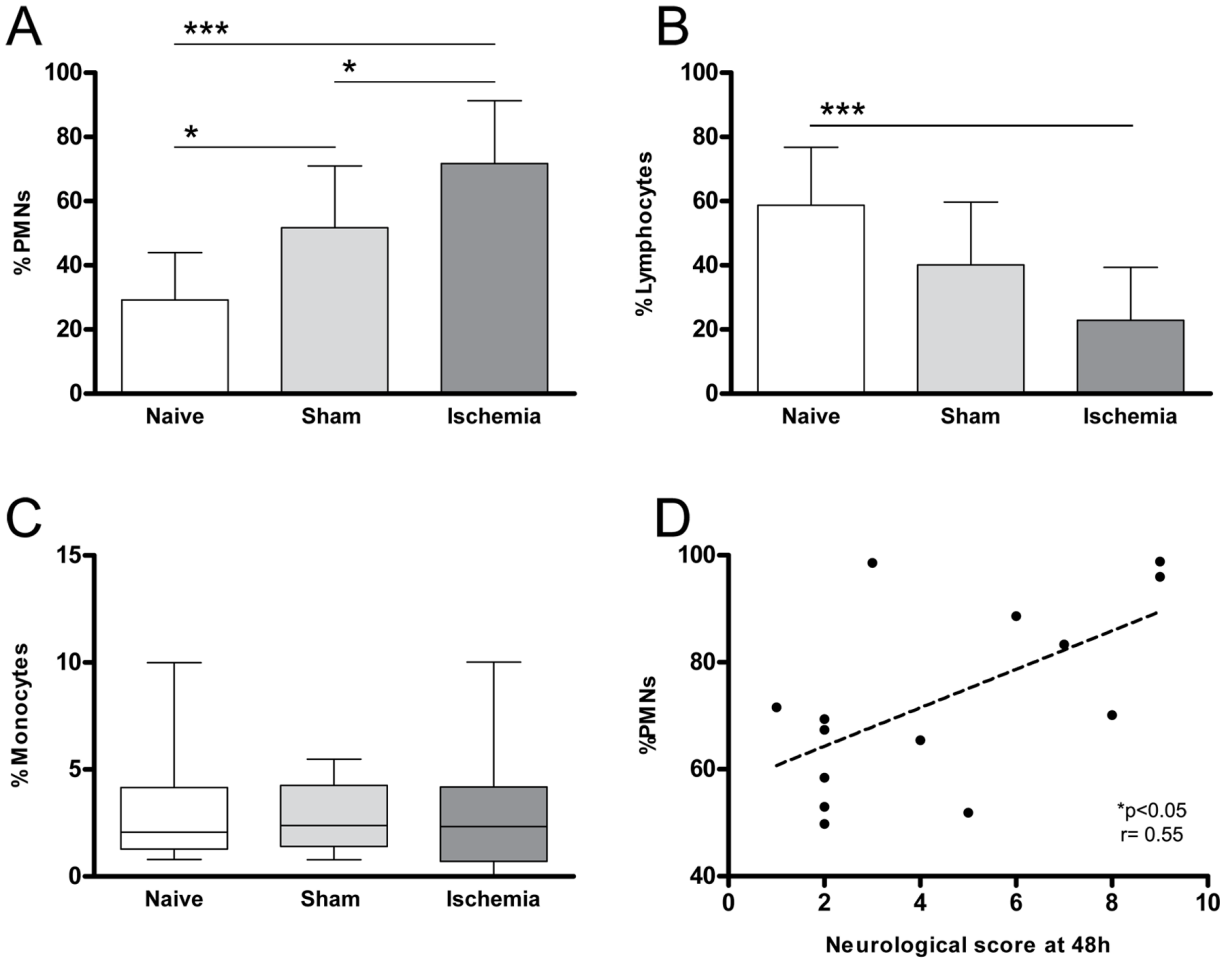
Leukocyte percentage assessed by flow cytometry of naive, sham or ischemic animals 48 hours after surgery. (A) Peripheral PMNs percentage: a significant increase was observed in sham and in ischemic animals with respect to the naive group (p<0.05). (B) Lymphocyte percentage. A significant decrease was detected between naive and ischemic animals (p<0.001) but no differences were shown between sham and ischemic animals. (C) Monocyte percentage. (D) Correlation between the percentage of systemic PMNs and the neurological score detected at 48 hours post-ischemia (p<0.05, r = 0.55). Naive (n = 9), sham (n = 13), ischemia (n = 14). *p<0.05, ***p<0.001.

Considering experimental groups, results obtained in flow cytometry assays are represented in Figure 2. Cerebral ischemia triggered an increase of PMNs in blood (71.7±19.5%) as compared to sham (51.7±19.2%) p<0.05; and naive rats (29.2±14.7%); p<0.01; (n =  9-14 per group) (Figure 2A) and conversely, decreased blood lymphocytes (22.90±16.4%) were found when compared either to sham (40.1±19.7%) or naive groups (58.8±18.1%), although only when comparing ischemic vs. naive the difference was significant, p<0.001 (Figure 2B). No differences in monocytes percentage were found among groups (naive: 2.07[0.79, 9.99] %; sham: 2.38[0.78, 5.48] %; ischemia: 2.32[0, 10.02] %), as shown in Figure 2C.

Interestingly, higher percentages of blood PMNs were positively correlated with a worse neurological outcome 48 hours after MCAO (p<0.05, r = 0.55); (Figure 2D), but no correlation was observed at other time points. Neither lymphocyte nor monocyte counts were correlated with neurological outcome.

### Bacteriological analysis

**Figure 3:**
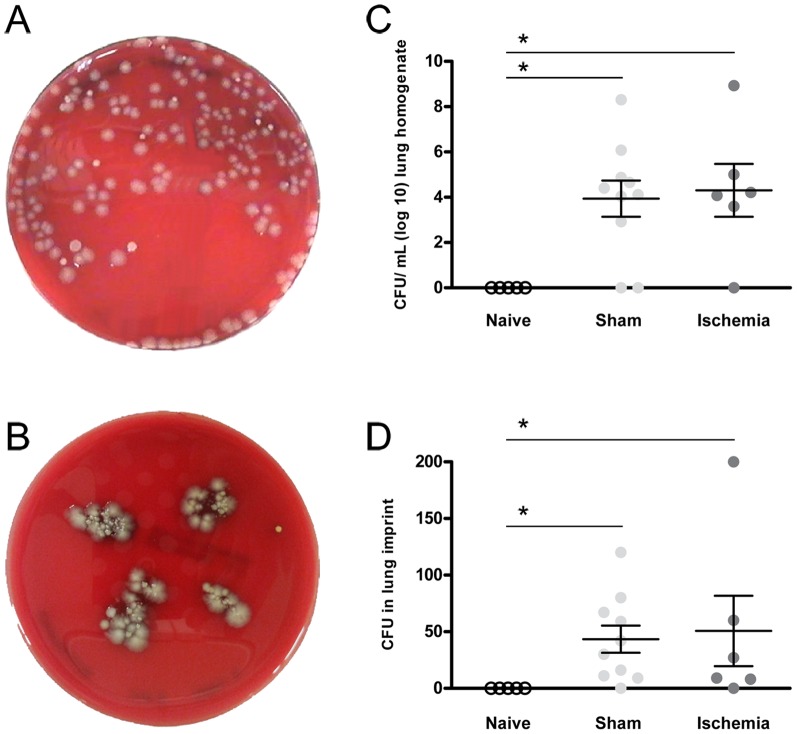
Bacterial growth 48(A) 200 µL of lung homogenate and (B) lung imprint. (C) Number of colonies (CFU) found in lung homogenates expressed as log 10. (D) Number of colonies in lung imprints. In the graphs, each dot represents an animal: naive (n = 5), sham (n = 10) and ischemia (n = 6).

Bacterial growth from lung homogenates plated on agar was detected in both sham and MCAO groups, as shown in figure 3A. In this sense, the number of CFU, was similar in sham rats (3.9±2.5; CFU/mL; log10) and in ischemic rats (4.3±2.9; CFU/mL; log10, p = 0.95) while naive lung homogenates did not show bacterial growth (Figure 3B). Contrarily, no CFUs were detected after plating blood samples of any of the groups. Regarding lung imprints (Figure 3C), CFU growth also appeared in both sham (43.40±38.09; CFU/mL) and ischemic (50.67±76.25; CFU/mL, p = 0.55) groups. However, no CFU were seen in naive lung homogenates, then indicating no previous pulmonary infection to the surgical procedure (figure 3D).

### Lung histology evaluation

**Figure 4:**
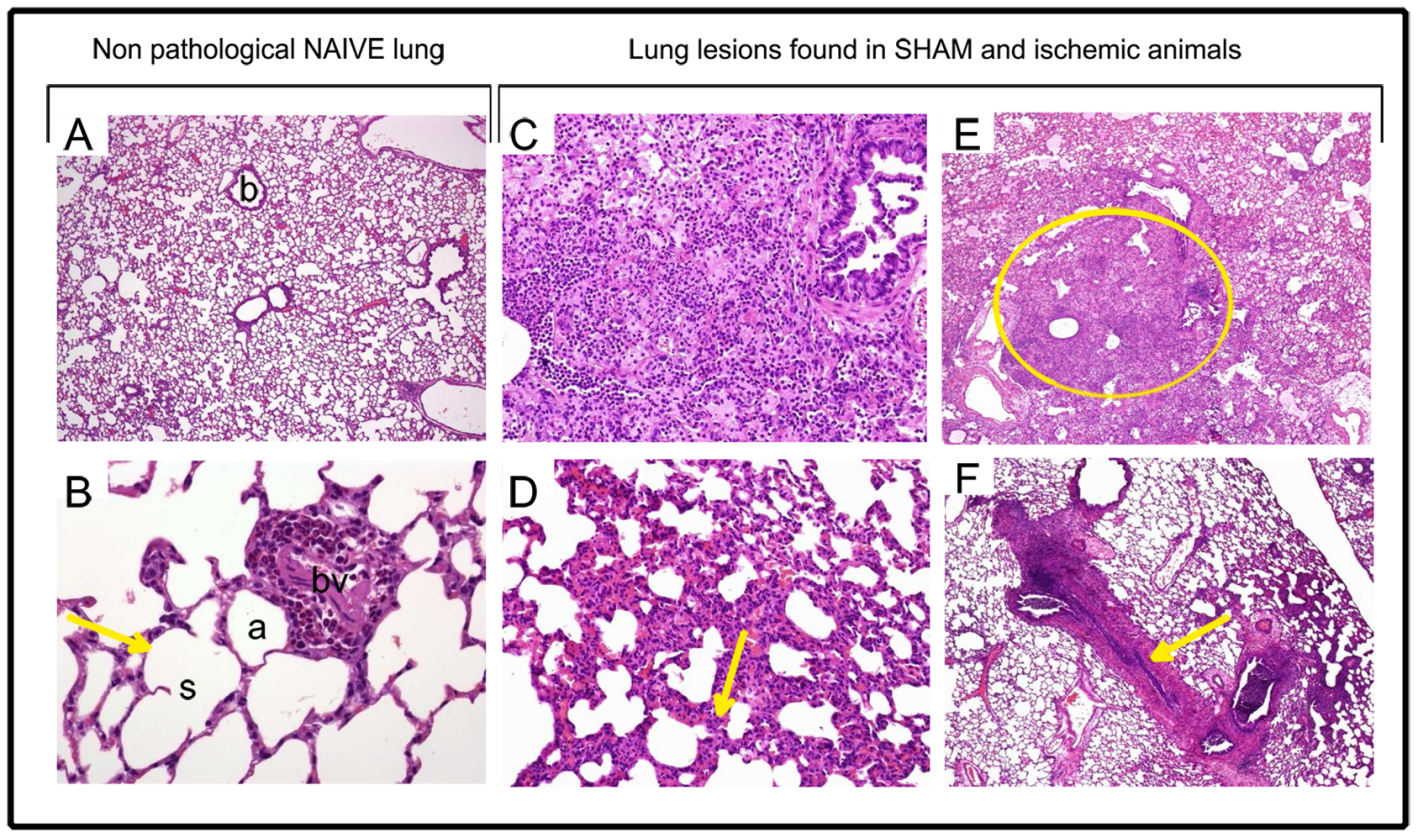
Representative images of lung histology after HE stain. (A) Naive rat lung showing a normal architecture (100x magnification), (b) indicates normal bronchioles. (B) Naive rat lung with alveolar sacs (s), expanded and non-filled alveoli (a), thin septa (arrow), and blood vessels (bv), (400x magnification). (C–F) Lung lesions observed in both sham and ischemic rats (C) Interstitial inflammatory cellular infiltration (100x magnification). (D) Thickened septa (arrow) and inflammatory cellular infiltration (400x magnification). (E) Patchy areas of cellular consolidation (circle), (100x magnification). (F) Acute bronchiolitis (arrow) surrounded by acute pneumonia (400x magnification).

As expected, all naive rats (4/4) showed normal lung histology consisting of definite alveoli with thin walls lined by two types of cells: pneumocyte type I, the predominant cell type, and pneumocyte type II, which are large and cuboidal and show short microvilli on their cell surface. The interalveolar septa appeared basically thin, although normal focal areas of thick septum could be observed. In lung tissue from naive rats we also observed that the BALT (bronchus-associated lymphoid tissue) which corresponds to the lung immune system, was generally located in bronchi bifurcation. (Figure 4A–B).On the other hand, both sham (n = 8) and ischemic (n = 8) groups showed altered lung histology involving acute vascular congestion (10/16), oedema (6/16) and intra-alveolar haemorrhage (4/16), in a diffused or patch distribution and affecting one or more pulmonary lobes. We observed lungs with lobular pneumonia and/or acute bronchopneumonia (4/16), characterized by PMN infiltration and associated to cellular necrosis and parenchymal fibrin inside alveoli or bronchioles. Some of these rats (3/16) showed also a mononuclear cellularity increase. In addition, in all these rats, although the number of BALT was strongly variable, they were always associated to segmented bronchi and without germinal centre, which is indicative of a reactive change (Figures 4C–F).

### Replication study using an intraluminal rat tMCAO model

Results obtained in this second set of experiments using a transient model with a nylon filament were equivalent to those derived from eMCAO model.The mean infarct volume of ischemic rats submitted to a tMCAO (n = 7) was 40.54±12.56%, and no lesion was detected in sham animals (n = 7). The mean neurological score was 4.14±1.07 for ischemic rats and 0 for sham rats.

**Figure 5:**
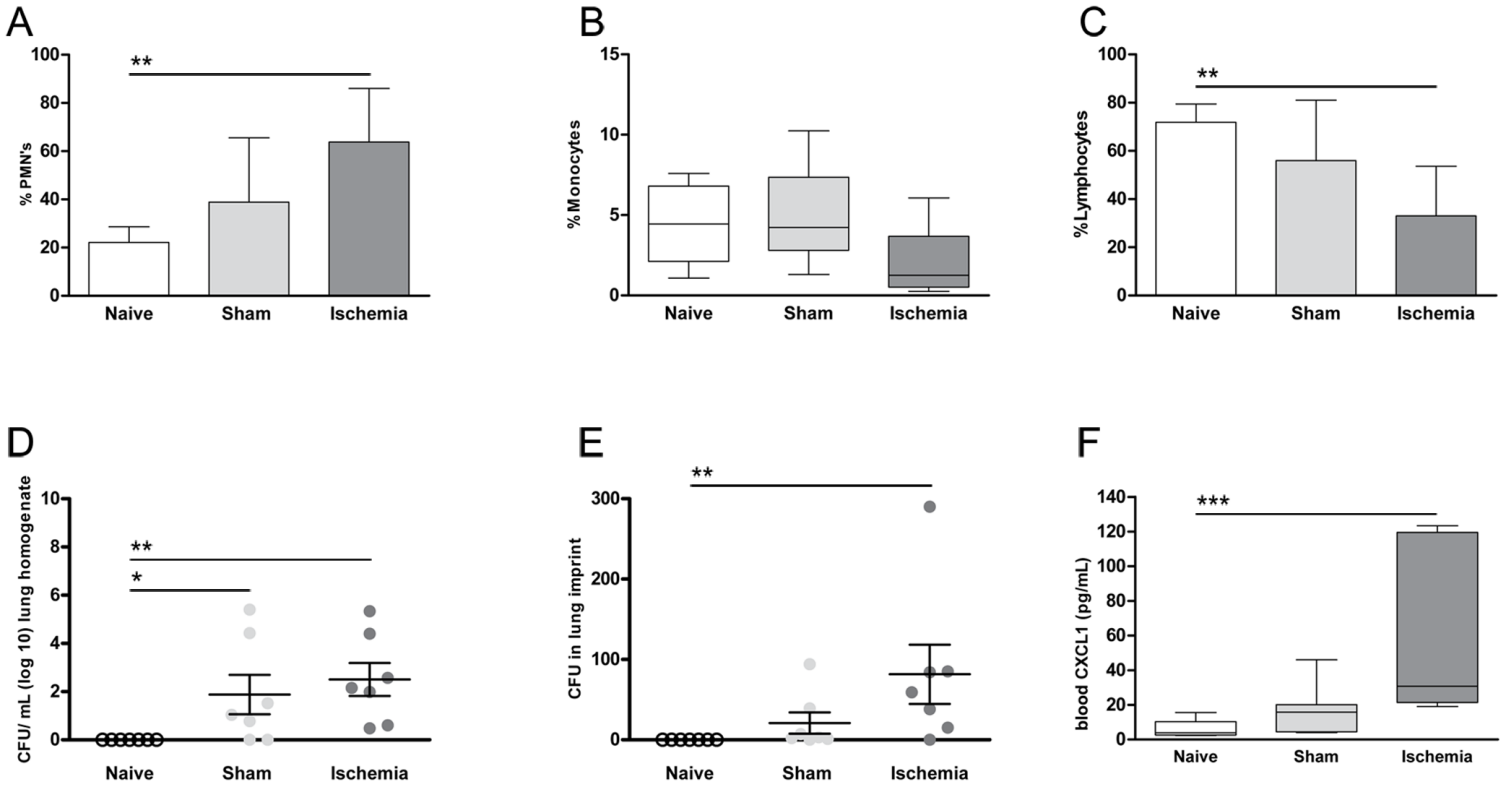
Leukocyte percentages, bacteriological analysis and measurement of CXCL1 plasma levels of a new set of animals submitted to a tMCAO model (90 min). Animals were evaluated at 48(A) Peripheral PMNs percentage: a significant increase was observed in ischemic animals with respect to the naive group (p<0.01). (B) Lymphocyte percentage. A significant decrease was detected between naive and ischemic animals (p<0.01) but no differences were shown between sham and ischemic animals. (C) Monocyte percentage. (D) Number of colonies (CFU) found in lung homogenates expressed as log 10. Significant differences were detected when comparing naive to sham groups (p<0.05) and naive to ischemia groups (p<0.01). (E) Number of colonies in lung imprints. Only the comparison between naive and ischemic animals reached significance (p<0.01). (F) CXCL1 plasma levels in naive, sham and ischemic rats. In agreement with peripheral PMNs percentage data, a significant increase was found in ischemic animals with respect to naive ones (p<0.001). In all graphs, naive (n = 7), sham (n = 7) and ischemia (n = 7).

Regarding blood leukocyte counts, we observed that ischemic animals showed an increase in blood PMNs (63.78±22.28%) as compared to sham (38.88±26.65%) or naive (22±6.5%) groups. In this case, only the comparison between naive and ischemic groups reached significance after Bonferroni correction (p<0.01) (Figure 5A). Also in agreement with the data previously obtained, a clear decrease in lymphocyte counts were found in ischemic animals (33.06±20.59%) when compared either to sham (55.94±25.11%) or naive animals (71.87±7.55%), being significant only the comparison between naive and ischemic groups (p<0.01) (Figure 5B). No differences in monocyte percentages were detected among the three groups (naive: 4.45 [1.08, 7.6] %, sham: 4.23[1.3, 10.24] %, ischemia: 1.25[0.25, 60.7] %) (Figure 5C).

In relation to bacteriological analysis, CFU growth was observed only after plating sham and ischemic lung homogenate samples. We could detect significant differences when comparing naive to sham animals (0 vs. 1.88±2.16 CFU/mL log10, p<0.05) and also naive to ischemic animals (0 vs. 2.5±1.81 CFU/mL; log10, p<0.01) (Figure 5D). No CFUs were obtained after plating blood samples of any of the groups. Considering lung imprints, we only found differences between naive and ischemic rats (0 vs. 81.57±97.43 CFU/mL, p<0.01), while sham animals showed a more moderate CFU growth (20.86±35.06 CFU/mL) (Figure 5E).

### CXCL-1 measurement

Plasma levels of CXCL1 were evaluated 48 hours after ischemia or sham surgery and also in naive rats (n = 7/each group). Sham animals showed upregulated levels (15.79 [4.02, 45.98] pg/mL) as compared with naive rats (3.9 [2.34, 15.53] pg/mL), although a more pronounced release was seen in ischemic animals (30.75 [19.16, 123.4] pg/mL). After correction, only significance was reached when comparing naive and ischemic animals (p<0.001) (Figure 5F). Correlation between PMNs mobilization and CXCL1 plasma levels was also evaluated and we found a clear significance (r = 0.66, p = 0.001) when animals of all groups were included (n = 28) and a trend towards significance (r = 0.71, p = 0.08) when only ischemic animals were evaluated (n = 7) (Data not shown).

## Discussion

Experimental stroke induces systemic inflammatory responses followed by global immunosuppression [7] that can make ischemic animals more prone to infections. In this study, we aimed to evaluate such stroke-induced infections using a MCAO rat model.

Blood analysis showed differences between sham and ischemic animals in circulating PMNs. According to previous studies, it has been well characterized that brain injury produces a secondary inflammatory response, accompanied by an increase on systemic neutrophils, the most representative PMN subtype [20], [21]. Interestingly, we observed a positive correlation between circulating PMNs and neurological deficit at 48 hours. In agreement with our results, other publications have reported an association between early elevation of systemic neutrophil count in stroke patients and ischemia severity [20]. On the other hand, similar to other publications performed with mice [13] or humans [8], [22], we detected a lymphopenia on our ischemic rats. Though we did not analyze lymphocyte subpopulations after stroke, we assume that it would have been of great interest as it seems to be a controversial issue. While most previous publications are in agreement showing a significant reduction in blood T-cells after stroke [23]–[26], the fact of whether or not B cells, NK cells, Tregs and monocytes are altered after cerebral ischemia is still on debate [26]–[29].

Remarkably, our results showed that leukocyte changes were in the same direction in both sham and ischemic groups. To explain this, we support that both isoflurane and sham surgery might disturb leukocyte responses, a hypothesis recently demonstrated in a published study [30]. This work reported that surgical stress and anaesthesia exert evident effects on both early and late cellular responses in the bone marrow after submitting mice to an experimental stroke. They also emphasized that volatile anaesthetics might affect leukocyte responses by altering neutrophil adherence to blood vessels and inducing lymphocyte apoptosis. Despite a significant decrease in lymphocyte percentage could be observed in ischemic compared to naive animals, no correlation was found considering the infarct size stated by others [31]. Our hypothesis is that all the disturbing factors entailed in these stroke animal models (anaesthesia, surgical stress, inflammation) may alter the association between infarct size and both the infection and the immunodepression severity.

Pneumonia and bacteraemia are the most common infections in stroke experimental studies [8], although bacteraemia appears less frequently and later than pneumonia. In human stroke, urinary tract infection has also a high incidence [3], [32] but it is not as common in animals as is in patients, probably because animals do not need to be catheterized. Regarding our bacteria colonies growth, surprisingly but in agreement with other studies [33], none of the animals have shown signs of blood infection. Contrarily, other studies detected bacteraemia after a transient MCAO in mice [8], [13], [14]. Although it has been reported that susceptibility to poststroke infections is species and strain dependent [34], we reckon that another explanation for the discrepancy in the results might be the short time our animals were under study before euthanasia (48 hours versus 3, 5 or 15 days reported by other studies). Furthermore, regarding our CFU/mL lung counts at 48 hours (4.3±2.9 CFU/mL log10), we consider they are in accordance with other publications [35] which reported around 3.5 CFU/mL log10 at 24 hours and 6 CFU/mL log10 at 72 hours after stroke in mice. However the variability in number and type of colonies found in different spontaneous infection studies has been demonstrated to be totally related with the environment where the animals are placed [33], [34].

Lung evaluation showed normal histology in naive rats whereas both sham and ischemic animals presented non-specific lesions such as distension of alveolar units, thickened alveolar septa, cellular consolidated areas, presence of inflammatory infiltrates, oedema and haemorrhage. The severity of these lesions was extraordinarily variable among animals of the same group in both sham and ischemic animals. Again, surgery stress, anaesthesia and inflammatory response due to the cranial trepanation could also explain these non-specific lung lesions found in both sham and ischemic animals.

Compiling our results, we have found many unexpected changes in sham animals in the present study. The animal (species and strain) or the model we have performed to induce ischemia could have had a big influence in our final results. To our knowledge, this is the first study inducing ischemia in rats by embolic MCAO and assessing bacterial analysis and lung histology thus we can speculate that the strong severity of the model (in part due to the cranial trepanation and the prolonged anaesthesia) could contribute to an important systemic inflammatory response. Despite our efforts in reducing the pain and discomfort of the rats subjected to an embolic stroke, the mortality associated to this experimental model is around 40%, as similarly shown in other studies [17] and can be attributed to the severe brain injury and to some secondary complications such as the loss of weight. Investigation of immunity and infection response at further time points (5–7 days after MCAO) would be, undoubtedly interesting. Nonetheless, such mortality rate during the first 48 hours limits the potential of studying longer time points that may allow us to assess more evident differences between sham and ischemic animals and avoid the effects of the surgery. Besides, although the embolic model is believed to be the most resembling stroke model to human pathology, it leads to a considerable variability among animals in terms of infarct volume and outcome which makes more difficult to reach to conclusions.

On the other hand, the fact of obtaining similar results after submitting the rats to a tMCAO model introducing a nylon filament and submitting them to an embolic MCAO model makes us consider the cranial trepanation and the cervicotomy as responsibles for the immunodepression state detected also in sham animals. In this direction, some authors concluded that even being sterile, brain surgeries may contribute to a general immunodepression [36] induced by stress and injury [37]. Considering this statement, our sham rats (submited to a cranial trepanation) could also be considered a model of brain surgery. Additionally, cervicotomy performed in both ischemic and sham animals, which can envolve iatrogenic manipulation of Vagus nerve, may also contribute to immune system alterations.

In agreement with Campbell *et al*. [38] who reported that after an acute injury in rodent brain, central nervous system triggers CXC chemokine expression (which is associated with leukocyte mobilisation), CXCL1 blood levels appeared elevated in both sham and ischemic animals in our study (although only the comparison between naive and ischemic animals reached significance). Moreover, good correlation was found between PMNs mobilization and CXCL1 blood levels. With these findings we can affirm; firstly, that both surgical stress and volatile anaesthesia exerted effects on chemokine response, as stated by Denes *et al*. [30]. Secondly, that cerebral ischemia *per se* triggers an additional chemokine effect. Thirdly, that in this animal model, as found previously in others [30], [36] chemokines such as CXCL1 are contributing to PMNs mobilization.

Consequently, an important result to highlight from our study is that including a sham animal group in stroke-induce infection studies is extremely important to identify surgery-related responses and avoid misinterpretation of the data from ischemic animals. Additionally, it might be of great importance to check the sanitary status of the animals (presence of opportunistic and not only pathogenic microorganisms) previously to the surgery if the aim of the study is to evaluate spontaneous infections.

In conclusion, our study could not discriminate ischemic from sham animals in terms of infection evaluation after embolic MCAO. Therefore, we cannot affirm that changes observed in ischemic animals are the consequence of the ischemic insult. The initial hypothesis of our study was that stroke-induced immunodepression in the rat would make ischemic animals more prone to respiratory infections. Considering very recent publications which report some neuroprotectants to ameliorate stroke-induced peripheral immunodepression [35], we thought that having a rat model of stroke-induced immunodepression would be of great importance to test potential drugs for immunomodulatory therapeutic strategies. But unexpectedly, we found that in almost all evaluated parameters, sham animals were more similar to ischemic than to naive animals. Thus, our caution is that both embolic and intraluminal nylon suture MCAO in rats are not suitable models to study infections after stroke. All in all, the differences on immunological parameters depending on the species and strain and the influence of anaesthetics and surgical stress have to be taken into account in further experimental and translational studies for immunomodulatory therapeutic strategies, at least in rat stroke models which entail cervicotomy and cranial trepanation.
